# Telehealth cancer care consultations during the COVID-19 pandemic: a qualitative study of the experiences of Australians affected by cancer

**DOI:** 10.1007/s00520-022-07021-6

**Published:** 2022-05-03

**Authors:** Victoria White, Alice Bastable, Ilana Solo, Seleena Sherwell, Sangeetha Thomas, Rob Blum, Javier Torres, Natalie Maxwell-Davis, Kathy Alexander, Amanda Piper

**Affiliations:** 1 School of Psychology, Faculty of Health, Deakin University, Geelong, VIC Australia; 2 Cancer Council Victoria, Melbourne, VIC Australia; 3 Loddon Mallee Integrated Cancer Service, Bendigo, VIC Australia; 4 Southern Melbourne Integrated Cancer Service, Melbourne, VIC Australia; 5 Bendigo Health, Bendigo, VIC Australia; 6 Goulburn Valley Health, Shepparton, VIC Australia; 7 Consumer Representative, Melbourne, VIC Australia

**Keywords:** Telehealth, Cancer, Qualitative research, Patient experiences, Carers

## Abstract

**Background:**

In response to the onset of the COVID-19 pandemic, telehealth was rapidly rolled out in health services across Australia including those delivering cancer care. This study aimed to understand people with cancer and carers’ experiences with telehealth for cancer care during the COVID-19 pandemic and associated restrictions.

**Method:**

Semi-structured interviews conducted with people with cancer and carers via telephone or online video link between December 2020 and May 2021. Participants were recruited through cancer networks and social media. Interviews were transcribed and thematic analysis undertaken.

**Results:**

Twenty-three patients and 5 carers were interviewed. Telephone-based appointments were most common. Responses to telehealth were influenced by existing relationships with doctors, treatment/cancer stage and type of appointment. Four themes were derived: (i) benefits, (ii) quality of care concerns, (iii) involving carers, and (iv) optimising use of telehealth. Benefits included efficiency and reduced travel. Quality of care concerns identified subthemes: transactional feel to appointments; difficulties for rapport; suitability for appointment type and adequacy for monitoring. Both patients and carers noted a lack of opportunity for carers to participate in telephone-based appointments. Aligning appointment mode (i.e. telehealth or in person) with appointment purpose and ensuring telehealth was the patient’s choice were seen as essential for its ongoing use.

**Discussion and conclusions:**

While telehealth has benefits, its potential to reduce the quality of interactions with clinicians made it less attractive for cancer patients. Patient-centred guidelines that ensure patient choice, quality communication, and alignment with appointment purpose may help to increase telehealth’s utility for people affected by cancer.

## Introduction

Telehealth or telemedicine involves the remote delivery of health services using technology including telephone and video [1, 2]. While Australia has utilised telehealth to deliver cancer care for over 20 years, its use has been largely restricted to patients living in rural and remote areas of the country [3]. The emergence of the COVID-19 (coronavirus-19) pandemic in 2020 saw countries across the world introduce measures to enable their health systems to manage the increasing numbers of people infected with the virus [1, 4, 5]. As part of these measures, the Australian government temporarily extended funding for telehealth consultations allowing for first-time specialist doctors, general practitioners, hospitals, and allied health professionals in metropolitan and regional areas of Australia to access this form of health care rather than use in-person appointments [6–8]. However, similar to other countries [9], the rapid implementation of measures relating to the COVID-19 pandemic in Australia meant there was little time for the development of protocols and training for delivering cancer care via telehealth [1, 10].

The impact of the rapid rollout of telehealth on patient experiences has been an area of many investigations, with a review finding 18 quantitative studies assessing patient experiences of telehealth were published in the first 6 months of 2020 [11]. This review found satisfaction with telehealth was generally high for people being treated by a range of services including oncology, mental health services, and sports medicine [11]. Most studies included in the review were from countries other than Australia and most reported on the experience of using video technology for appointments [11]. Studies published subsequently have continued to demonstrate high levels of satisfaction with telehealth for outpatient appointments in mixed patient populations, with video [12–14] and telephone [15] formats. However, despite high satisfaction, multiple studies have also shown that most patients prefer in-person consultations [16] suggesting that a more nuanced understanding of telehealth experiences is needed.

Although government policy for telehealth in Australia recommends video over the telephone for these appointments [1], most consultations in 2020 utilised the telephone, with one study suggesting 57% of all telehealth appointments in the second half of 2020 were by telephone [1] and another finding that 82% of appointments with cancer specialists in June 2020 were over the telephone [7]. Australian patients also report that the telephone has mainly been used for telehealth appointments [17, 18] with this also true for cancer appointments [10, 19]. Despite the greater use of the telephone, Australian surveys have shown that patients generally accept telehealth, appreciating it as a strategy for reducing risks during the COVID-19 pandemic [10, 17, 20, 21]. However, the lack of a physical examination [1, 22] and concerns regarding its impersonal nature are noted as barriers and reasons why many patients say they will return to in-person appointments [10, 20].

Due to the long-term nature of much cancer care, the bond and trust many patients develop with their clinical team can contribute to the therapeutic alliance many see as a cornerstone of patient-centred medical care [23]. As a mutual trust, respect, and agreement on therapeutic goals are key characteristics of the therapeutic alliance [24], it is important to understand the impact of telehealth on cancer patients’ care experiences especially if telephone rather than the video is used to deliver most of these appointments. This study aims to understand Australian cancer patients’^1^ experiences with telehealth and to examine the pros and cons of this appointment type in the delivery of cancer care. Recognising the role that partners and carers play in the care of many cancer patients [25–28], this study expanded its focus to assess how carers experienced telehealth. This study used a qualitative methodology to explore the telehealth experiences of cancer patients and carers from the Australian state of Victoria during the COVID-19 pandemic.

## Method

### Design

Qualitative study consisting of semi-structured interviews with 28 people affected by cancer (*n* = 23 patients and *n* = 5 carers) who had at least one telehealth consultation with a member of the cancer care team in 2020. Data collection stopped when data saturation was achieved.

### Context

Participants for the study were residents of Australia’s second most populous state, Victoria. Victoria especially its capital city Melbourne, had the longest period of lockdown and social restrictions in the country. Interviews for the study were conducted between December 1, 2020, and May 15, 2021. While during most of this period, restrictions to reduce the spread of the virus were eased, a short lockdown (5 days) was introduced in Melbourne in February 2021, with another starting at the end of May 2021.

### Ethics

The research had ethical approval from Deakin University (reference HEAG-H 203_2020).

### Participants

Eligibility criteria were diagnosed with cancer, or cared for someone with cancer, in the previous 5 years, attended a medical appointment delivered via telehealth for cancer care in 2020/21, aged over 18 years and could speak and read English.

### Procedure and data collection

Participants were recruited through patient and supporter networks associated with cancer advocacy and support organisations and through networks associated with health services. Interested participants were directed to an online site where they accessed information about the study and provided their consent for study participation. Participants scheduled an interview time online after registering consent. Semi-structured telephone interviews were conducted using an interview guide developed by the research team. The telephone was the preferred method for undertaking the interview as it allowed greater flexibility for participation. While the option was available to participants to use an online video call system, only two interviews (each involving a patient and carer) were conducted this way. Interviews lasted approximately 30 min. All interviews were conducted by the same female behavioural researcher (VW) who has a PhD, over 20 years’ research experience in supportive care interventions, cancer management and cancer prevention, and experience in undertaking qualitative research with people affected by cancer. Notes on the interview were recorded afterwards. Participants had no prior relationship with the researcher. Participants were given a brief overview of the researcher’s reasons for the research. Transcripts were not returned to participants for comment or correction. 

### Interview schedule

The interview included questions on participants’ cancer and the type of care they were receiving during 2020 (treatment, follow-up) and then moved on to assess participants’ experiences with telehealth for their cancer care. Questions assessed the type of technology used (telephone/video), number of telehealth appointments, information received about the appointment, difficulties with the technology, communication challenges, pros and cons of telehealth for cancer care, and thoughts regarding the continued use of telehealth. Questions were derived from previous work by team members assessing cancer patients’ telehealth experiences. The investigator team reviewed and finalised the interview schedule.

### Analysis

Transcripts and recordings were reviewed, and a thematic analysis was conducted [29]. Microsoft Excel was used to manage data. Analyses were undertaken by VW and ST. An iterative process was used with VW undertaking the initial review of transcripts to develop a draft coding structure based on themes and subthemes emerging from the data and ST independently reviewing transcripts to confirm the coding structure. Differences were discussed (ST and VW), and the coding structure was refined to reflect consensus decisions. Codes were grouped into broad thematic areas and identified themes discussed with the research team to ensure clarity and consensus.

Reporting follows the COREQ guidelines [30]. Participant quotes are presented to illustrate themes with quotes from patients noted with the code P and patient interview number (e.g. P1) and quotes from carers noted with the code C and a carer interview number (e.g. C1).

## Results

### Sample

The sample included 13 males and 15 females. All five carers involved in the study were female (Table 1). Participants with cancer commonly had prostate (*n* = 5), breast (*n* = 5), or ovarian (*n* = 3) cancer. Of the carers interviewed, three were partners of men with prostate cancer. Most participants (*n* = 19) were under 70 years of age, lived in metropolitan areas (*n* = 21), and were treated in the public ﻿﻿﻿health system (*n* = 21). Fifteen patients indicated the telephone was used predominately for their telehealth appointments and four mainly had video consultations (Table 1). Mostly, participants spoke of telehealth appointments with a single health professional, with only one carer describing a video consultation with a multidisciplinary team of health professionals to discuss treatment planning. While participants indicated telehealth appointments with a range of different doctors (e.g. medical oncologists, haematologists), only four patients mentioned telehealth appointments with allied health professionals usually physiotherapists or psychologists.

**Table 1:** Participant cancer, health system, sex, residential location, and format for telehealth characteristics

Characteristic		*N*
Participant type	Patient	23
	Carer	5
Sex	Male	13
	Female	15
Residential location	Metropolitan	21
	Regional	7
Health system	Public	21
	Private	7
Cancer type (patients)	Prostate	5
	Breast	5
	Ovarian	3
	Lung and mesothelioma	3
	Blood	2
	Other	5
Cancer stage in 2020	Treatment	8
	Follow-up/monitoring	15
Telehealth format	Telephone	18
	Mix	6
	Video	4

### Telehealth experiences

For all participants, the response to telehealth was influenced by several factors particularly their relationship with the doctor, their treatment and cancer stage, the type of information delivered in the appointment, and the level of vulnerability felt in relation to their health. While the technology used for the appointment played a part in some participants’ responses, a greater influence seemed to be participants’ relationship with their doctor and the type of appointment. While a theme of ‘benefits’ was derived from the discussion of pros associated with telehealth, the discussion of cons was more substantive reflecting concerns about the ‘quality of care’ received through telehealth. Two other key themes were identified from the interviews: ‘including carers’ and ‘optimising use of telehealth’. The themes are discussed in detail below.

### Benefits

Most participants acknowledged the benefits of telehealth with these mainly focusing on improved convenience due to reduced waiting and travel times and ease of access when not feeling well (see Table 2 for exemplar quotes). While some noted that being able to have the appointment from the comfort of home meant less disruption to activities, another noted that telehealth provided greater flexibility as she was not tied to one location for appointments. Others noted that the continued medical oversight and reduction in COVID-19 infection risk was a key benefit. One carer noted that due to her partner’s declining health and mobility, telehealth reduced the stress associated with in-person appointments particularly in relation to travel, time out of the day, negotiating wheelchairs in and out of cars, and parking.

**Table 2:** Exemplar quotes relating to the theme of benefits of telehealth

Subtheme	Exemplar quote
Reduced travel	Convenience of not having to travel to [hospital] and paying a $20.00 parking fee. (P11)
	I have to allow a lot more time for actually getting there and getting home [for in person appointments]. There is also the cost factor, both the economic and time costs. (P2)
	If it [telehealth] works that’s a great way for anyone who’s a long way away from the hospital because you have other anxieties as well. Things like having to go through peak hour traffic, it can be rather wearing if you’re not use to it. That’s one of the benefits of telehealth because you don’t have to do that. You don’t have the build up of tension, which is one good benefit, there is also no waiting time. (P2)
Reduced costs	Financially advantageous, because it was bulk billed. (P13)
	We never got a bill for them [telehealth appointments]. (P15)
Reduced downtime/stress	If they are running late, you’re in your own home so you can keep pottering around chipping away at all the jobs that need to be done. So you don’t feel like you lose huge portions of your day like you do when you’re there in person. … The removal of travel time which gives you back more time in your day. But also because [my husband] is full time in a wheelchair, it’s hard work getting into the car and driving into the hospital and getting him to the doctors rooms. (C1)
Convenient if not feeling well	It’s also very helpful when you’re feeling sick and tired because the last thing you want to do is get out of bed or get off the couch. You know you’re going to vomit or you’re going to have diarrhea from the chemo, so you don’t want to be in a car for half an hour. A lot of times those mornings I was grateful that I could do a telehealth. (P3)
Kept me safe	They [telehealth] worked really well and kept me safe. (P10)

## Quality of care concerns

Comments relating to the negatives of telehealth reflected concerns around ‘quality of care’. Four subthemes were identified: (i) transactional; (ii) difficult for rapport; (iii) suitability for appointment type, and (iv) adequacy for monitoring. Exemplar quotes are shown in Table 3 for themes discussed below.

**Table 3:** Exemplar quotes for subthemes in the ‘quality of care concerns’ theme

Subtheme	Exemplar quotes
Transactional	I feel the phone is just a bit too quick and not treated like a real appointment. (P7)
	Its not particularly patient centric, pretty transactional (P13)
	I wouldn’t say it was rushed but it felt incomplete. I got to the end of the phone call and they knew a bit more about me, but I didn’t know what the next steps were. And it is hard to get a feel for what an organisation is like, when you are doing it over the phone. (P5)
	There was no rapport, no nothing, it was just business (P3)
	You have to have a list of questions that you want to ask because it’s very easy to forget until after you finished the call, “I should have said this, or I should have said that. So I think you have to go in prepared. (P6)
	What I have found with the telephone consultation they tended to be, not superficial, but they didn’t really encourage going into depth. (P2)
	But if you as a patient can’t say to your doctor: Look, I felt really sick yesterday, I was feeling better today and the reason I’m feeling that way is because this, this and this. You’ve got to be able to say that to them regardless. It doesn’t matter whether they’re on the phone or whether you’re sitting in front of them. (P26)
	No real difference because I have a good relationship with the doctors anyway. I tend to research things so if I have a question, I can raise it. (P14)
Difficult for rapport	I found them adequate but they were just adequate….. I would have preferred video rather than telephone, because I find that having the visual connection as well as the verbal connection adds to the discussion. (P2)
	I think that is the thing, trying to get a rapport with a doctor you don’t know is pretty impossible over the phone…..(P5)
	I thought that telehealth delivers a great service, but it was very impersonal at first. (P10)
Suitability for appointment type	It [cancer diagnosis] was the worst thing anyone could possibly say to us so it’s a lot easier for us if someone was looking at us and explaining what our situation is in a face to face manner, than over the phone. (C4)
	I would be quite happy to go with telehealth but if I had more pressing problems I would probably opt for face-to-face. (P12)
	I prefer face to face, unless it’s just for a script or something, I would use it to renew a script. It's important to have that face to face [consultation] (C3)
Adequate for monitoring?	I didn’t have an examination for the year of COVID and that did cause me some concern even though my CA 125 is so low. So I think any doctor would say it’s fine, not having examination. But it did cause me some concern (P9)
	When you walk into a surgery, doctors are taking your appearance in, when they talk to you on the phone or looking at you on video they don’t get a real picture of what you are actually doing, how you are looking. They don’t get an accurate picture of you as a person and what is actually happening. They don’t see you get out of a chair, drink a cup of coffee. (P10)
	When I had a face-to-face consultation, I was able to have a much more thorough conversation, which bought out an issue that was concerning me to do with bowel function which led me to have a colonoscopy which then found pre-cancerous polyps. (P13)

### Transactional

For those having telehealth via a telephone, the most common description of their experiences was that it was ‘transactional’, quick, and more business-like than face-to-face appointments. While these descriptors were less commonly used for video appointments, even these were seen as less engaging and quicker than face-to-face appointments. A number of participants commented that telehealth appointments had a different quality to them with one feeling they did not encourage going deeply into conversations with the doctor, while another indicated it was easier to ‘brush someone off’ in a telehealth appointment. One participant who mainly had telephone appointments noted that from a philosophical position, a telehealth appointment was a ‘less symbolic’ activity. This participant saw the physical act of going to a medical appointment to meet with the ‘healer’ as part of the ritual of treatment. Existing relationships with doctors influenced responses, with those having a good ongoing relationship with their doctor reporting no difference in connecting with their doctor via telehealth regardless of whether it was via video or telephone.

Many participants noted that they needed to be more prepared and proactive in asking questions in telehealth appointments, particularly telephone-based appointments. This was due to both doctors being less able to assess body language, and appointments being quicker. However, patients’ general approach to asking questions influenced comments, with some noting that they tended to do their research and ask questions at appointments regardless of format. Others noted that doctors differed in their tendency to ask questions with telehealth exacerbating the situation when doctors didn’t usually ask many questions.

### Difficulties for rapport

Many participants commented on the difficulty of developing rapport with doctors via telehealth especially when their first contact with a doctor was via telehealth. This was partly due to telehealth appointments lacking the incidental conversations people have with their doctors. One participant noted that when telehealth was used for the first appointment with a clinician or hospital, it made it difficult to get a sense of the organisation, which led them to feel less certain of the care they were receiving.

### Suitability for appointment type

There was a strong response from participants that some appointments were not suitable for telehealth with these including receiving a diagnosis or bad news, treatment planning, meeting a doctor for the first time, and when a physical exam was needed. A carer reported that her husband received his cancer diagnosis over the phone with no preparation, no follow-up, and no support. The carer keenly felt the inappropriateness of using telehealth in this instance.

Appointments that were suited for telehealth included regular routine check-ups (if occurring more frequently than once a year) and script renewal.

### Adequacy for monitoring

While the lack of a physical examination was noted as a limitation, comments suggested this was only one aspect of feeling monitored. The lack of visual contact when phones were used created concerns regarding adequate monitoring of health. People discussed how doctors use body language, including how someone walks, how they dress and how they respond to questions, to form assessments of health and function. The absence of different visual cues created concern that health issues would be missed. Participants noted that the lack of incidental conversations could also limit their doctor’s ability to identify potential health issues. Several participants reported previous experiences where an incidental conversation or their doctor’s interpretation of their physical appearance, led to tests or investigations that identified further health issues. Comments suggested concern that these health issues would have been missed with telehealth.

## Involving carers

Many patient participants noted that telehealth made it more difficult for their carers to participate in appointments. They noted that they were never asked if a carer wanted to attend an appointment and while reflecting that they could put the call on speaker if the carer wanted to participate or invite the carer to the video call, this was made difficult if the carer had not set time aside for the appointment. Patients noted that carers often helped to remember questions as well as the content of appointments; without carers, patients felt questions could be more easily forgotten (see Table 4 for exemplar quotes).

**Table 4:** Exemplar quotes relating to themes: ‘Where do carers fit?’ and ‘optimising telehealth’s inclusion’

Theme	Exemplar quote
Involving carers	I feel I need to be at appointment to ask the questions so it's very hard, with telehealth for us both to be there at the same time. It’s easy to say “I can't come to work today because I'm going to the hospital then to say “I need time off [for telehealth consultation] because it’s like a phone call” (C4)
	The biggest problem that I have with it is that my husband couldn’t come into my treatment consultations with me. He has been with me from the beginning at every consultation but in the telehealth ones of course he’s not necessarily around to be involved. He has missed that particularly because I have to relay information to him second hand. And sometimes you forget what you want to tell the doctor …quite often he would back me up and say you have forgotten to tell her [doctor] about such and such. Quite often your carer will come up with questions that I hadn’t thought of, and I think that is what we miss more than anything with the telehealth. (P11)
	A couple of times I pop them on speaker phone so my friend can hear. My friend noticed that I was forgetting a lot of information that they would tell me, so I needed a second pair of ears. (P3)
	The doctor, especially when I do these video-conference things they should be aware that carers could be there. So I think the doctor or the specialist should be saying ‘is your wife/husband there?’ and they should bring them into the conversation straightaway, instead of me saying ‘my wife is here’ (P18)
Optimal inclusion of telehealth	There’s probably a bit of criticism around telehealth, but I think it’s it is a really great format. Really something that should be rolled out. (P1)
	I know it’s subsidized by the government, but I think the option should be there for [telehealth] to be kept on, to give people the option. (P18)
	I think video health that would be, that’s the only way I can do it. I don’t think I’d do telephone. (P1)
	video would be better [then telephone] because it’s nice to see who you are speaking to (P6)
	Making sure that the health system gives people the option of what type of appointment they want, but also providing people with the information about when is a good time for a telehealth appointment versus when a face-to-face appointment is needed (P11)
	“The opportunity for telehealth or maybe every second or third visit be a face to face so that you can still build that rapport with your clinician. (P21)
	I think it should be a mix of telehealth and face to face contact, because you have to go in for your scans. Not losing physical contact is important, often you think about things on the spot or you can have a question answered. I like the telehealth, but you need a mixture of both. If people like physical examinations, they shouldn’t be doing telehealth. If it’s just about the return of results I think you could do that on telehealth. (P2)
	It would help if they gave some instructions on how it’s going to go, this would also help your partner. ….. Using telehealth there were no instructions on how the meetings were going to run or what was going to happen. You didn’t know if your partner could be there or anything like that. (P8)

Carers had mixed experiences of telehealth. Carers with more positive experiences tended to have video appointments where they were included in appointments simply by sitting next to the patient for the consultation. Negative experiences related to telephone-based appointments as well as the type of appointment/stage of care. One regional carer reported her terminally ill husband was only offered telephone appointments; this excluded her from appointments and restricted information flow regarding his care needs.

## Optimising the use of telehealth

Most patients and carers thought telehealth had a place in their ongoing care but wanted to ensure it was there to benefit patients (Table 4 for exemplar quotes). All participants noted the importance of patients choosing when to have a telehealth appointment with these decisions needing to consider appointment type, familiarity with the doctor, and time since the last in-person appointment. Patients and carers considered blending telehealth with in-person appointments was ideal as this would ensure personal connections could develop with health professionals while enhancing convenience when in-person appointments were not needed.

## Discussion

Similar to other countries [31–33], Australia rapidly expanded the use of telehealth in response to the COVID-19 pandemic to ensure access to health care while reducing demands on hospitals and limiting social interaction [1]. However, despite recommendations that telehealth appointments make use of video technology, the majority of telehealth appointments in Australia were conducted via the telephone [1, 7]. The current study identified a range of responses to telehealth in Australians affected by cancer, which included recognising benefits along with limitations including appointments feeling more transactional and generating concerns regarding the adequate monitoring of their health. While these limitations were found regardless of the technology used, they were most evident for telephone-based consultations. The inclusion of carers in telehealth appointments was also seen as difficult with telephone appointments again presenting greater problems. As all participants expressed interest in maintaining telehealth as an option for their health care, protocols and procedures need to be developed to address concerns regarding the quality of care [34].

Responses to telehealth were influenced by perceptions regarding the quality of care. Previous work has noted that the lack of a physical examination is a key limitation to telehealth reducing perceptions of care quality [9, 10, 35]. While this concern was evident in our data, this was not the only factor influencing the quality of care perceptions: the lack of visual contact with telephone-based appointments and limited visual contact with video appointments also influenced concerns. Patient participants were aware that doctors use many different tools to assess their health including body language, gait, and information gathered through incidental conversations and were concerned that these cues were missing in telehealth. Other Australian studies have found that patients have less confidence in doctors’ ability to monitor health with telehealth [10, 17, 19]. While some international studies have suggested that the lack of physical contact and limited visual cues in telehealth present challenges to health professionals [15, 36], other studies suggest that doctors compensate for this in video consultations by increasing their use of and attention to body language [36]. Australian patients’ concerns regarding the adequacy of their monitoring through telehealth may reflect the use of the telephone and unfamiliarity with these appointment types.

Like others [10, 35, 37], we found satisfaction with telehealth depended on whether there was an existing relationship with the clinician and the reason for the consultation. When appointments were with a familiar doctor and for check-ups, they were satisfactory, although perhaps more ‘business-like’. Others have noted the different ‘feel’ of telehealth consultations with one study reporting patients see them as ‘cold and impersonal’ [20] and a review finding the perception of these appointments as impersonal a barrier for health professionals [38]. In our study, telehealth appointments were frequently described as transactional or business-like, suggesting that the more patient-centred or personal aspect of appointments was missing. An early study looking at patient-doctor communication in video-based telehealth appointments compared to in-person appointments found that video consultations were more doctor-centred with patients asking fewer questions [39]. Most appointments cancer patients have will be clinician initiated, with clinicians having a clear understanding of what they want to assess/monitor in appointments. To reduce the transactional feeling of telehealth appointments, developing clinicians’ skills in assessing and responding to verbal and nonverbal cues patients provide during a telehealth appointment will be important. Studies have suggested that patients can feel connected and can develop a rapport with doctors when using video- or telephone-based telehealth [14, 33, 40, 41]. Training for health professionals regarding communication skills for use in telehealth appointments will be important to ensure ongoing quality of care for patients [42].

Carers in our study had a range of experiences with some finding the reduction in travel and associated difficulties with transporting high-needs patients beneficial, while others found them impersonal and lacked support. Response of carers often related to the type of appointment telehealth was used for and how easily they were included. Replicating experiences with in-person appointments, patients reported they were not asked if they wanted to have someone with them during their telehealth appointment. However, while carers can just attend in-person appointments, their inclusion in telehealth appointments depended on the technology used, with video more inclusive than the telephone. A survey study of Australian cancer patients and carers also noted the difficulties carers faced in attending telephone-based telehealth appointments [10]. Similar to findings from the current study, that study also found that the exclusion of carers from appointments made it more difficult for them to provide the same sort of support to patients. In contrast, work from New Zealand suggests that moving to video-based telehealth made it easier for the family to attend appointments and be involved in care [37]. More work is needed to understand the experiences of carers in telehealth and to develop effective strategies to ensure they can be included in appointments when appropriate.

Optimal cancer care involves a team approach to care with relevant health care professionals (including supportive care professionals) reviewing and discussing treatment options and management plans [43]. In our study, few participants discussed appointments involving multiple clinicians or those delivered by nurses or allied health. Further work is needed to explore the use of telehealth for nursing or allied health consultations and when consultations involve more than one health professional.

Our study has a number of limitations that must be noted. We recruited people through social media posts, support groups, and email lists associated with large well-known cancer support and advocacy organisations. This approach may have meant our sample was technology and internet literate. While we spoke to a range of people with cancer, our sample of carers was relatively small and mainly limited to carers of prostate cancer. Others have noted difficulties with recruiting informal carers into research projects with low involvement associated with the carer’s gender, preference to focus on patient welfare, and lack of time [44]. As we recruited participants through patient and cancer organisations’ supporter networks informal carers may not be as connected to these networks reducing their exposure to study information. As our results suggest telehealth delivered via a telephone has a substantial impact on the involvement of carers in cancer care further work focusing on carers is needed. As interviews were conducted in English, the experiences of those from other linguistic and cultural backgrounds need to be explored.

The expansion of telehealth during 2020 allowed cancer patients access to their health care team while reducing risk of exposure to COVID-19. While telehealth is associated with many benefits, these were balanced against concerns regarding its potential negative impact on the quality of care. Greater access to video technology for telehealth appointments and availability of technological support for clinicians and consumers to ensure adequate access may alleviate some concerns and assist with the inclusion of carers. Our findings strongly support recommendations that clinical appropriateness and patient choice are the foundation principles in the design of telehealth systems and the development of guidelines for its use [8]. They also support the need for training health professionals in how to communicate and build supportive environments for patients via telehealth [42].

## Data Availability

The data that support the findings of this study are available from the corresponding author on reasonable request. The data are not publicly available due to privacy and ethical restrictions.

## Footnotes

1 The authors recognise that people with cancer are people first and foremost rather than a ‘patient’. However, for the purpose of being concise, we refer to people with cancer as ‘patients’.
